# Evolution of *cagA* Oncogene of *Helicobacter pylori* through Recombination

**DOI:** 10.1371/journal.pone.0023499

**Published:** 2011-08-11

**Authors:** Yoshikazu Furuta, Koji Yahara, Masanori Hatakeyama, Ichizo Kobayashi

**Affiliations:** 1 Department of Medical Genome Sciences, Graduate School of Frontier Sciences, University of Tokyo, Minato-ku, Tokyo, Japan; 2 Institute of Medical Science, University of Tokyo, Minato-ku, Tokyo, Japan; 3 Graduate School of Medicine, Kurume University, Kurume, Fukuoka, Japan; 4 Fujitsu Kyushu Systems LTD, Fukuoka, Fukuoka, Japan; 5 Department of Microbiology, Graduate School of Medicine, University of Tokyo, Bunkyo-ku, Tokyo, Japan; 6 Department of Biophysics and Biochemistry, Graduate School of Science, University of Tokyo, Minato-ku, Tokyo, Japan; Veterans Affairs Medical Center (111D), United States of America

## Abstract

*Helicobacter pylori* is a gastric pathogen that infects half the human population and causes gastritis, ulcers, and cancer. The *cagA* gene product is a major virulence factor associated with gastric cancer. It is injected into epithelial cells, undergoes phosphorylation by host cell kinases, and perturbs host signaling pathways. CagA is known for its geographical, structural, and functional diversity in the C-terminal half, where an EPIYA host-interacting motif is repeated. The Western version of CagA carries the EPIYA segment types A, B, and C, while the East Asian CagA carries types A, B, and D and shows higher virulence. Many structural variants such as duplications and deletions are reported. In this study, we gained insight into the relationships of CagA variants through various modes of recombination, by analyzing all known *cagA* variants at the DNA sequence level with the single nucleotide resolution. Processes that occurred were: (i) homologous recombination between DNA sequences for CagA multimerization (CM) sequence; (ii) recombination between DNA sequences for the EPIYA motif; and (iii) recombination between short similar DNA sequences. The left half of the EPIYA-D segment characteristic of East Asian CagA was derived from Western type EPIYA, with Amerind type EPIYA as the intermediate, through rearrangements of specific sequences within the gene. Adaptive amino acid changes were detected in the variable region as well as in the conserved region at sites to which no specific function has yet been assigned. Each showed a unique evolutionary distribution. These results clarify recombination-mediated routes of *cagA* evolution and provide a solid basis for a deeper understanding of its function in pathogenesis.

## Introduction

*Helicobacter pylori* is a pathogenic bacterium that colonizes the stomach of at least half of the world's human population [1]. It is known to cause gastric diseases including gastritis, gastric ulcer, duodenal ulcer, and gastric cancer. Many virulence factors are involved in its pathogenicity, and its genome is known for high geographical diversity [2].

Cytotoxic antigen gene A (*cagA*) is one of the most studied of the pathogenicity genes of *H. pylori* [1]. The gene *cagA* is on a genomic island, or the cag pathogenicity island (cagPAI), which is hypothesized to have been acquired by horizontal gene transfer [3]. CagA protein is injected into host epithelial cells by a Type IV secretion system encoded by cagPAI. In the host cells, CagA is localized to the inner membrane and phosphorylated by host SRC family kinases [4]. Phosphorylated CagA binds to host proteins such as SHP2, CSK, and PAR1. Binding to SHP2 activates an intracellular signalling pathway that lies downstream of ligand-stimulated growth factor/cytokine receptors, leading to cytoskeletal rearrangement [4], [5]. Binding to PAR1, which has a central function in the establishment and maintenance of the basolateral membrane [6], [7], leads to loss of epithelial polarity [8], [9].

**Figure 1:**
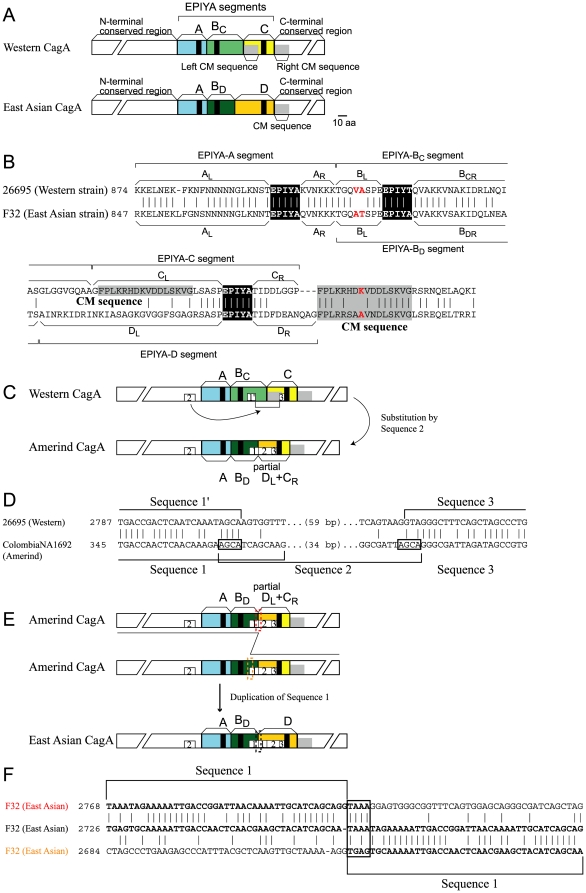
CagA protein and its evolutional pathway. (A) CagA typical of Western strains and East Asian strains. (B) Aligned amino acid sequences of a Western CagA (strain 26695, hpEurope) and an East Asian CagA (strain F32, Japanese). Red: positively selected amino acid changes. Black: EPIYA motif. Gray: CM sequence. (C) Organization of each type and proposed steps of evolution. (D) Alignment of sequence 2 substituted region between Western type and Amerind type. (E) Alignment of sequence 1 duplicated region between Amerind type and East Asian type. Sequences presumed to be involved in recombination are boxed. (F) Alignment of sequence 1 duplication.

CagA is known for variability in its C-terminal region, which includes a motif of five amino acid residues: glutamic acid-proline-isoleucine-tyrosine-alanine, designated an EPIYA motif (Figure 1A, B). The tyrosine residue is the target of phosphorylation by SRC family kinases [4]. The EPIYA motif is found in four types of EPIYA segment classified by sequence similarity into EPIYA-A, EPIYA-B, EPIYA-C and EPIYA-D. In many *H. pylori* isolates in Europe, North America, and Australia, CagA carries EPIYA-A, EPIYA-B and EPIYA-C segments in tandem and is called Western type. CagA from East Asian countries such as Japan, Korea and China carries EPIYA-A, EPIYA-B and EPIYA-D segments in tandem and is called East Asian type (Figure 1A, B). Evolutionary relationships of the Western and the East Asian types remain unclear. SHP2 strongly binds to the EPIYA motif in the EPIYA-C and EPIYA-D segments, which carry a SHP2-binding motif around the EPIYA motif. The East-specific D segment shows higher binding affinity to SHP2 than the West-specific C segment [10]. East Asian *cagA*-positive *H. pylori* infections are more closely associated with gastric cancer [11] and an East Asian type CagA can induce tumors in mice more efficiently than can Western CagA [12], [13].

The EPIYA-C segment carries a 16 amino acid sequence at its N-terminal end, termed a CagA Multimerization (CM) sequence or a Conserved Repeat responsible for Phosphorylation-Independent Activity (CRPIA) motif (grey in Fig. 1A, B) [14], which was originally identified as the sequence that mediates multimerization of CagA for stable binding with SHP2 protein [15]. The CM sequence is repeated just downstream of the EPIYA-C segment (Fig. 1A, B).

Although the majority of *cagA* found in *H. pylori* isolates encodes three EPIYA segments, ABC or ABD, many structural variants have been reported (for review, see [16]). The number of EPIYA segments can vary from one to seven [17]. Some *cagA* alleles encode more than two EPIYA-C segments, which can provide more sites for SHP2 binding. There are also chimeric EPIYA segments made of two segment types [17], [18]. These structural variants may have been generated by a recombination mechanism.

*H. pylori* is known for high rates of mutagenesis and homologous recombination, which may be related to absence of mismatch recognition [19], [20]. There should occur homologous recombination, which use long nearly identical sequences as targets [21], [22], and illegitimate recombination, which involves short related sequences [23]. Homologous recombination between allelic DNA sequences is common because of high natural competence [19]. DNA transposons often insert themselves into the genome with short target duplication, while restriction-modification systems sometimes insert themselves with long target duplication [24], [25]. Several site-specific recombination enzyme homologs occur in *H. pylori* genomes [26]. DNA duplication is sometimes associated with inversion [27]. Variation in the length of simple repeats leads to phase variation [28].

In this study, we aimed to understand the evolution of the *cagA* sequences through recombination mechanisms. We compared the nucleotide sequences of all the available *cagA* sequences. We were able to explain their relationship using three modes of recombination and to propose a clear evolutionary route to the East Asian type.

## Results

### Classification of *cagA* structural variants by recombination mechanisms

All CagA amino-acid and *cagA* nucleotide sequences were retrieved from NCBI databases (see Materials and Methods), and entries with the entire variable C-terminal region were selected (1118 entries) (Table S1).

CagA entries were assigned to segment types by BLASTP search [29]. Many of them fell into two typical organizations, EPIYA-AB_C_C (Western type, 324 entries, 29%) or EPIYA-AB_D_D (East Asian type, 524 entries, 47%). The extent of each segment was as defined previously (Figure 1B) [10]. In addition to the terms “EPIYA motif” to represent the EPIYA sequence and “EPIYA segment” to represent regions including the sequence, we designated the two parts of each segment flanking EPIYA motif (often abbreviated as Ψ here) by L (left) and R (right) subscripts (Fig. 1A, B). Thus, an A segment is expressed as A_L_ΨA_R_, B_C_ as B_L_ΨB_CR_, B_D_ as B_L_ΨB_DR_, C as C_L_ΨC_R_, and D as D_L_ΨD_R_. (We did not classify the left half of the EPIYA-B segment into B_CL_ and B_DL_ because their sequences are very similar in the East Asian (B_D_) and Western (B_C_) strains. For example, there is 22/24 nucleotide sequence identity and 6/8 amino acid sequence identity between Western 26695 strain and East Asian F32 strain.) Note that Ψ indicated a collection of EPIYA motifs that does not necessarily have the amino-acid sequence exactly EPIYA. Note also that EPIYA and similar symbols may indicate nucleotide sequences (in *italic*) as well as amino-acid sequences (in roman). We sometimes use *EPIYA* to specifically indicate a nucleotide sequence (or nucleotide sequences) corresponding to the EPIYA motif. Likewise, a CM sequence may indicate an amino-acid sequence or a corresponding nucleotide sequence. We may use *CM* to specifically indicate the nucleotide sequence.

By analyzing amino-acid and nucleotide sequence alignments, we realized that all structural variants of *cagA* could be explained by three modes of recombination: (i) homologous recombination at the *CM* sequence; (ii) recombination at the *EPIYA* sequence; and (iii) recombination between short similar sequences. All structural variant types are presented in Figure S1, and all *cagA* entries are classified in Table S1. These are explained in turn below.

### Generation of the left arm (D_L_) of East Asian-specific EPIYA-D segment from Western *cagA*

The EPIYA-D segment, which is specific to East Asian CagA, contains some of the mysteries of the *cagA* gene, specifically when, where, and how East Asian CagA emerged. Its development cannot be explained by accumulation of mutations in the *EPIYA-C* segment because of the extent of divergence between the *EPIYA-C* and *EPIYA-D* segment. Now, we found that establishment of *D_L_* can be explained by a two-step intragenic rearrangement from Western type *C_L_*.

**Figure 2:**
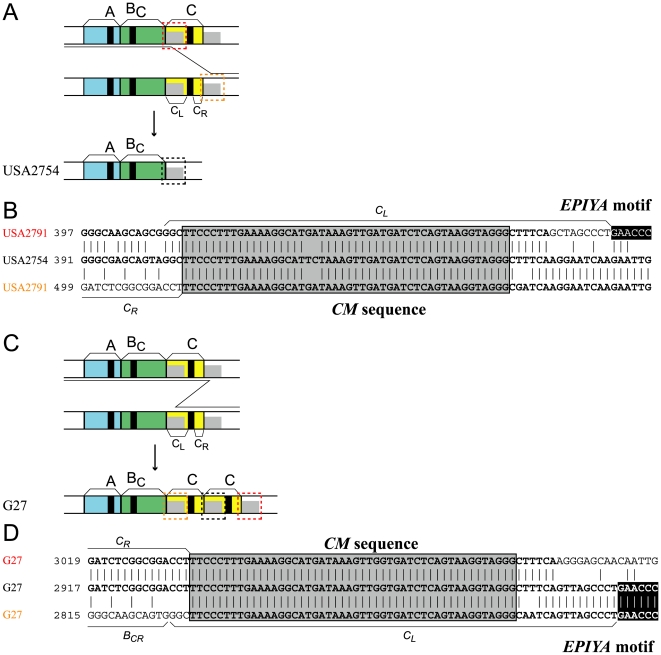
Copy number variation of EPIYA segments by homologous recombination at the *CM* sequence. (A) Deletion of the EPIYA-C segment. (B) DNA sequence alignment. (C) Duplication of the EPIYA-C segment. (D) DNA sequence alignment. Black: *EPIYA* motif. Gray: *CM* sequence.

*D_L_* can be divided into three sequences: 1, 2, and 3 (Fig. 1C, E). Surprisingly, Sequence 2 has significant similarity (approximately 60% in nucleotide sequence identity) with a sequence encoding the N-terminal conserved region of *cagA* (Fig. 2A, B, C). East Asian *cagA* has two copies of Sequence 1 in tandem, whereas Amerind *cagA* has only one copy. The Western type has only the 5′ half (represented as sequence 1′) in *B_CR_* (Fig. 1C, D). Taking these gene structures into consideration suggested a hypothetical evolution pathway to *D_L_* as follows.

First, a copy of Sequence 2 replaced the right end of *B_C_* and the *CM* sequence of *C_L_* in the Western *cagA*, thereby generating the Amerind *cagA* (Fig. 1C). This process created the intact Sequence 1, which consists of Sequence 1′, and the 5′ half of Sequence 2 (Fig. 1C, D). Next, tandem duplication of Sequence 1 occurred in the Amerind *cagA*, generating East Asian type *cagA* (Fig. 1E). A four-bp sequence, 5′ TAAA, is the probable site for the illegitimate recombination leading to the duplication (Fig. 1F). This site is not included in Sequence 1′ of Western *cagA*, so this amplification must have occurred after the Sequence 2 substitution.

We are, however, aware that an alternative route of recombination between the duplicated Sequence 1 leading to loss of one copy cannot be formally ruled out by this sequence comparison. In any case, the above-presented model points to the notion that the left arm of the *EPIYA-D* segment (*D_L_*) can simply be generated through intragenic recombination events in Western *cagA*. Given this fact, we argue that Amerind *cagA* is the intermediate between the Western *cagA* and East Asian *cagA* based on parsimony.

### Homologous recombination between *CM* sequences

Some Western strains lack the EPIYA-C segment and show a pattern of AB (18 entries, 2%, Fig. 2A, B). This loss can be explained by apparent unequal recombination between the two 48-bp *CM* sequences, which were reported before the definition of the CM sequences [30]. The two sequences are sufficiently long and similar for homologous recombination, based on other prokaryotes [21], [31]. The recombination may have taken place within a genome or between incoming homologous DNA and a resident genome. The latter is known to be frequent in *H. pylori* [19].

Increase of the C segment occurred in Western strains, resulting in up to five copies. We observed EPIYA-ABCC (114 entries, 10%), -ABCCC (20 entries, 2%), -ABCCCC (2 entries, 0.2%), and -ABCCCCC (3 entries, 0.2%). The increase can be explained by unequal recombination between two *CM* sequences (Fig. 2C, D).

Consistent with this mechanism, the D segment in East Asian CagA, which lacks a CM sequence (Fig. 1A, B), cannot duplicate. For duplication of the entire *EPIYA-D* segment, *cagA* must adopt mechanisms that do not require long homologous sequences (discussed below). Lack of *CM*-mediated recombination might have accelerated diversification of the East Asian CagA CM sequence from the Western CagA CM sequence [32].

Our analysis of consensus sequences by LOGO [33] revealed that these CM sequences are highly conserved at the nucleotide and amino-acid level (Fig. S2A, B). This is consistent with homologous recombination. A specific recombination breakpoint was not identified.

### *EPIYA*-specific recombination

Chimeric EPIYA segments consisting of sequences of different types, instead of the major EPIYA segment types (A, B_C_, B_D_, C, D), have been reported [17], [30]. In this work, 81 entries (7%) were observed to have at least one such chimeric segment (Table S1). Our detailed analysis at the nucleotide sequence level revealed that generation of these chimeric segments could be explained by recombination at a breakpoint within the 15-bp DNA sequence encoding the EPIYA motif, which we designated as *EPIYA*-specific recombination. The concepts of *EPIYA*-specific recombination follow the paradigm of site-specific recombination [34].

**Figure 3:**
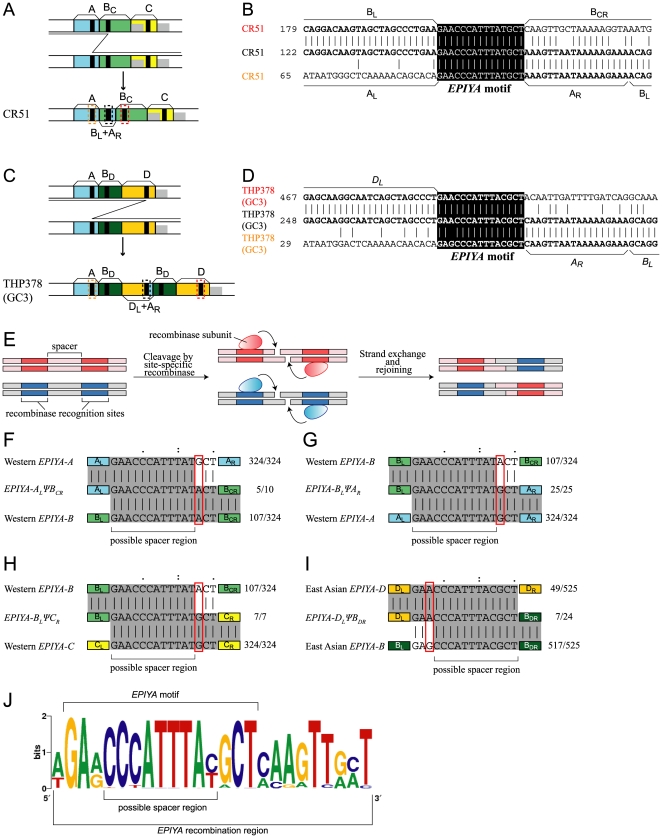
Generation of a chimeric EPIYA segment by recombination between *EPIYA* motifs. (A) A chimera of EPIYA-B_L_/EPIYA-A_R_. (B) DNA sequence alignment. (C) A chimera of EPIYA-D_L_/EPIYA-A_R_. (D) DNA sequence alignment. Black: *EPIYA* motif. (E) General scheme of site-specific recombination. Modified from Fig. 11-4 in [60]. (F–I) Estimation of possible spacer region within *EPIYA* motif. (J) Consensus sequence around the *EPIYA* motif.

Among all the possible 15 chimeric combinations of the left and right parts of the CagA EPIYA segments, 12 are found in the database (Table 1). For example, B_L_ and A_R_ paired in strain CR51 (Fig. 3A, B) and D_L_ and A_R_ paired in strain THP378 (GC3) (Fig. 3C, D). The formation of some of the chimeric segments requires recombination between a Western type *cagA* and an East Asian type *cagA*: specifically, the combination of EPIYA-C segment and EPIYA-D segment. C_L_ΨD_R_ was observed in a Japanese strain, F80 [18], and the D_L_ΨC_R_ combination was observed in all isolates from native Americans in Peru [35], Venezuela [36] and Colombia [37], which were reported earlier as products of unusual *cagA* alleles [35], [36].

**Table 1:** Numbers of major and chimeric EPIYA segments.

Left/Right	A_R_	B_CR_	B_DR_	C_R_	D_R_
A_L_	1110 (31)^a^	10 (0.3)	8 (0.2)	1 (0.03)	0 (0)
B_L_	24 (0.6)	512 (14)	588 (16)	7 (0.2)	2 (0.06)
C_L_	3 (0.08)	4 (0.1)	0 (0)	686 (19)	2 (0.06)
D_L_	1 (0.03)	0 (0)	23 (0.6)	10 (0.3)	581 (16)

a : Percentage of 3572 *EPIYA* segments, within 1118 *cagA* sequences.

Compared to the *CM* sequence for homologous recombination, the *EPIYA* sequence is only 15-bp long, which is too short for homologous recombination at least in other organisms [21]. Hence, recombination at *EPIYA* probably occurs through site-specific recombination using the activity of a site-specific recombinase that might recognize the DNA sequence around and/or within *EPIYA* and induce DNA strand breakage and rejoining at the spacer region between those recognition sites (Fig. 3E).

To identify the location on the DNA strand of the breakage and rejoining events, *EPIYA* sequences that likely underwent the recombination were compared to those without, or before, the recombination. The latter were extracted from major Western *cagA* genes (*EPIYA-ABC*) and major East Asian *cagA* genes (*EPIYA-ABD*) (Fig. S2C–H). Several differences were observed between the *EPIYA* sequences (Fig. S2C–H; Fig. 3F–I).

For example, in the major Western *cagA*, the 13th nucleotide is always G in the DNA sequences for *EPIYA-A* (324/324) and *EPIYA-C* segments (324/324), whereas the same position is mixture of A (107/324), G (216/324), and T (1/324) in the sequences for *EPIYA-B* segment. The 13th nucleotide of the DNA sequence in *EPIYA-A_L_ΨB_CR_* is a mixture of A (5/10) and G (5/10), which suggested that the nucleotide sequence to the right of the 13th nucleotide is already part of the sequence corresponding to *EPIYA-B* segment, and the recombination occurred to the left of the 13th nucleotide (Fig. 3F). This is also supported by the observation that the 13th nucleotide for *EPIYA-B_L_ΨA_R_* is always G (25/25) (Fig. 3G), and that for *EPIYA-B_L_ΨC_R_* is always G (7/7) (Fig. 3H). These 13th nucleotides show the same nucleotide frequency as the right half of the hybrid, corresponding to the *A_R_* and *C_R_* segments.

In the prototype East Asian *cagA*, the third nucleotide is almost always G for the *EPIYA-B* segment (A, 8/524; G, 516/524), whereas the same position is a mixture of A and G for EPIYA-A (A, 49/524; G, 473/524; T, 2/524), and *EPIYA-D* (A, 466/524; G, 58/524) segments (Fig. S2). The third nucleotide of the *EPIYA* sequence in *EPIYA-D_L_ΨB_DR_* is a mixture of A (7/24) and G (17/24), suggesting that the nucleotide sequence to the left of the third nucleotide is still a part for the *EPIYA-D* segment (Fig. 3I). Hence, the recombination breakpoint is probably at least after the fourth nucleotide. Taken together, we conclude that the break/rejoin point likely lies between the 8th and the 12th nucleotides of the *EPIYA* sequence for EPIYA motif.

When the sequences around *EPIYA* that have experienced recombination were compared, a weak consensus was found at the one-bp upstream site, and the nine-bp downstream region (Fig. 3J). Thus, this 25-bp (1-bp+15-bp+9-bp) region may include recognition sites by the putative recombinase and its cofactors, if any, as well as a spacer region. This region was designated as *EPIYA* recombination region.

In several cases, recombination between an *EPIYA* sequence and a *CM* sequence led to partial deletion of the *CM* sequence (Fig. S3). We found that the *CM* sequence includes a sequence with a weak similarity to the 9-bp sequence downstream of *EPIYA* mentioned above (Fig. S3B–D). Therefore, this recombination may have been erroneously mediated by the putative *EPIYA*-specific recombination machinery. On the other hand, we cannot exclude the possibility that the *CM-CM* homologous recombination might somehow involve this 9-bp sequence.

### Recombination between short similar sequences

**Figure 4:**
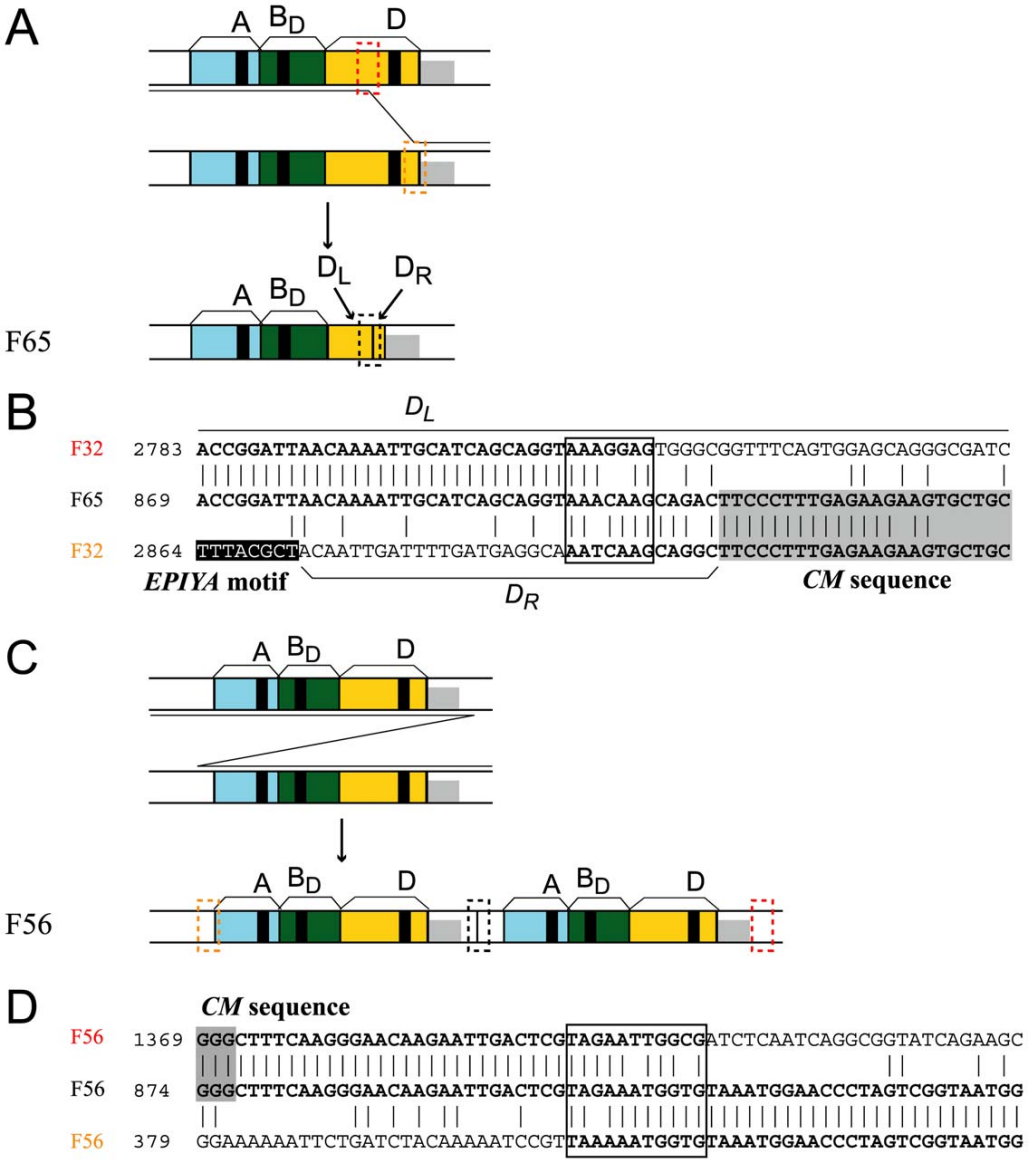
Rearrangement by illegitimate recombination between two short similar sequences. (A) Deletion of an EPIYA motif. (B) DNA sequence alignment. (C) Duplication of the entire EPIYA region. (D) DNA sequence alignment. Sequences presumed to be involved in recombination are boxed. Black: *EPIYA* motif. Gray: *CM* sequence.

Formation of the *cagA* variants that could not be explained by the above two modes of recombination could be explained by illegitimate recombination between short (1 to 12-bp) similar sequences (Fig. 4, Fig. S4, Table S1). For example, in strain F65, illegitimate recombination at the 7-bp sequences similar to 5′ AAACAAG, both upstream and downstream of the *EPIYA* sequence in *EPIYA-D* segment, explains deletion of the EPIYA motif (Fig. 4A, B). On the other hand, the entire A-B-D region was duplicated in F56, likely by illegitimate recombination at the 11-bp 5′ TAGAAATGGTG sequences upstream of the *EPIYA-A* segment and downstream of the *CM* sequence (Fig. 4C, D).

### Positive selection for amino acid changes

Positively selected amino-acid changes were searched throughout the *cagA* gene (Table S3). We detected seven amino acid residues (Table 2): two within the EPIYA-B_L_ segment, one within the CM sequence (red in Fig. 1B), and the other four in the N-terminal and C-terminal conserved regions, which have been rarely studied. This result suggests the presence of functional residues in these conserved regions.

**Table 2:** Sites for adaptive amino acid changes in CagA protein.

Position in		Residue in 26695	
Alignment	26695		Comment
98	82	T	N-terminal conserved region
687	634	E	N-terminal conserved region
1219	910	V	EPIYA-B_L_
1220	911	A	EPIYA-B_L_
1724	989	K	CM sequence downstream of EPIYA-C or D
1866	1111	V	C-terminal conserved region
1917	1160	T	C-terminal conserved region

**Figure 5:**
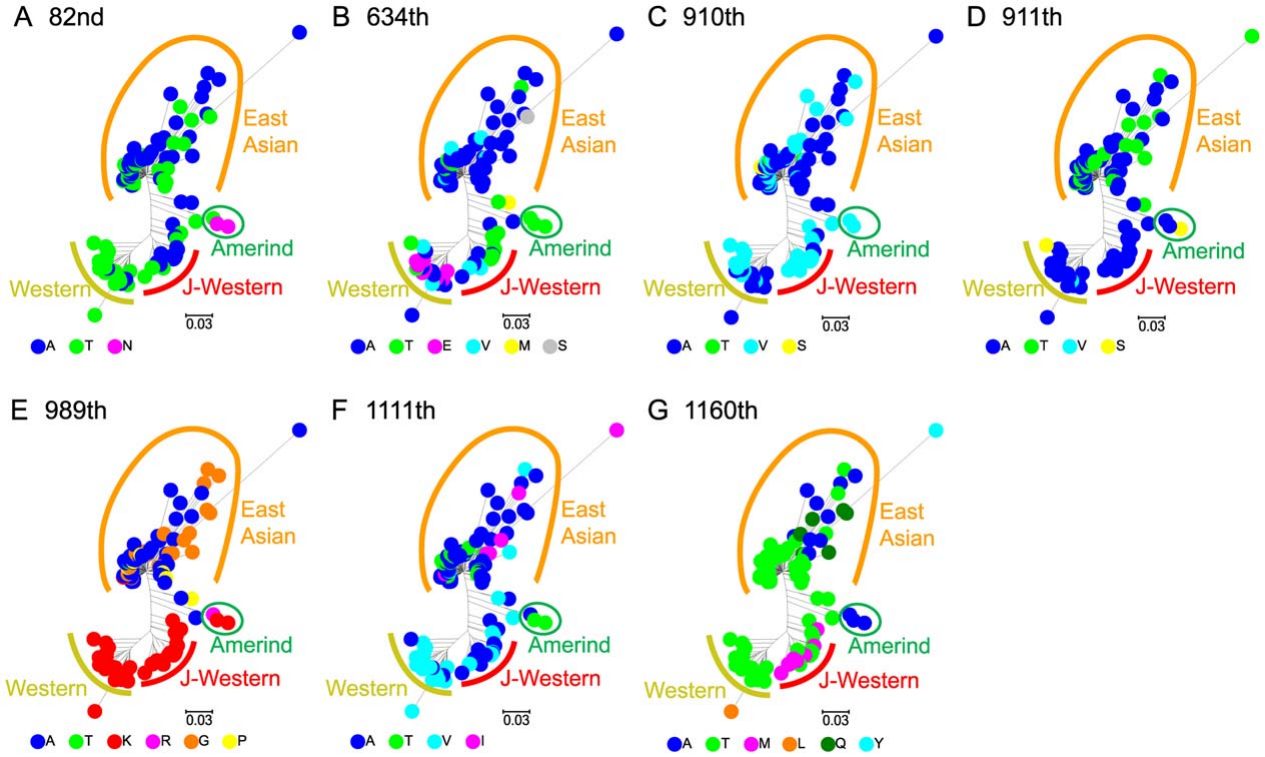
Amino acids at sites with positive selection for change on the *cagA* phylogenetic tree. The tree is based on nucleotide sequences of the entire gene. Each codon number refers to those in strain 26695. (A) 82nd codon. (B) 634th codon. (C) 910th codon. (D) 911th codon. (E) 989th codon. (F) 1111th codon. (G) 1160th codon.

These amino acid residues were plotted on a nucleotide-based phylogenetic tree of the entire *cagA* gene to study the time and mode of the positive selection (Fig. 5). Nodes of the tree were grouped into four: Western, J-Western, Amerind and East Asian [38], [39]. The J-Western group mainly consists of Okinawa strains from several southern islands in Japan [11].

Some codons showed a specific pattern that suggested positive selection for adaptation to a geography-specific environment. For example, the residue in the CM sequence (989th in strain 26695) showed a clear separation between the East Asian and Western/J-Western CagA (Fig. 5E). The residues in the right CM sequence are reported to interact with PAR1/MARK kinase [32], [40], [41]. Therefore, the amino acid changes there might reflect difference in interaction of CagA with the kinases with selective advantage between the two groups.Residue 634 (in strain 26695) is diverse within each of the three groups, Western, J-Western and East Asian (Fig. 5B), and may have experienced diversifying selection within each group. At the 1160th codon in strain 26695, a methionine residue was observed only in the J-Western group (Fig. 5G). This suggested unique evolution of this codon in this group.

## Discussion

In this study, we examined the recombination processes underlying the wide variation in the *H. pylori cagA* gene through DNA sequence alignment of all *cagA* sequences. We classified the underlying processes into three mechanisms: (i) homologous recombination at the *CM* sequence; (ii) recombination at the *EPIYA* sequence; and (iii) illegitimate recombination between short similar sequences. Some alleles appear to have experienced two of these processes (Table S1, Figure S4). Our results also revealed a plausible evolutionary pathway to the East Asian *cagA*.

Frequency of the *cagA* variants of each class is roughly consistent with the frequency of each recombination mechanism, as expected from the length of sequence similarity. Homologous recombination at the 48-bp *CM* sequence occurred in 30% (161/529) of Western strains. Recombination at the 25-bp conserved sequence around the *EPIYA* sequence occurred in 6.4% (34/529) of Western strains and 6.9% (40/581) of East Asian strains. Illegitimate recombination at 1 to 19-bp sequences occurred in 1.5% (8/529) of Western strains and 3.1% (18/581) of East Asian strains. This tendency is consistent with previous reports (for example, [21]). This simple relationship is impressive because the abundance of a particular genotype is a result of both formation and selection.

### Molecular mechanisms of *cagA* recombination

Among the three mechanisms, recombination by the *CM* sequence can be explained by homologous recombination because of the length of the sequence identity (48-bp). *H. pylori* is known for a high recombination rate [19].

In the second process, recombination occurred specifically around the *EPIYA* sequence, so that this might be the result of site-specific recombination by enzymes such as integrases, resolvases, and invertases [42]. Several integrases are found in the *H. pylori* genomes, such as *xerCD* homologs on TnPZ [26], an integrase homolog in the prophage region [43], and a serine-type recombinase on IS*607* [44] and IS*Hp609* [45].

Other enzymes may be involved in the recombination. IS*606* is reported to insert itself next to 5′ TTTAT or 5′ TTAT, and IS*608* is reported to insert itself next to 5′ TTAC [46]. These sequences are in the 8th to 12th nucleotides in the consensus sequence of *EPIYA*, so transposases of these IS elements could mediate recombination at *EPIYA* sequences. The transposase homologs may catalyze site-specific recombination, as seen for Neisserial Nf bacteriophage families [47].

For the third case, illegitimate recombination between short similar sequences, no consensus sequence was found. Although the target sequences seem to be AT rich, no common pattern was observed. This process might occur during replication or recombination, as proposed for illegitimate recombination in other organisms.

### Evolution of the EPIYA-D segment via Amerind CagA

Here, we provide a possible evolutionary pathway for the *EPIYA-D* segment by sequence rearrangement within the *cagA* gene. We suggested that the Amerind *cagA* could have been the intermediate between the Western and East Asian *cagA*. If this indeed was the case, Amerind *cagA* must have arisen before the crossing of Bering Strait to generate *H. pylori* strains carrying the EPIYA-D segment in East Asia. Then, why is the Amerind *cagA* yet to be found in East Asian countries? Has it disappeared through selection or genetic drift? Traces of Amerind *cagA*, if any, might be obtained from further sampling of *H. pylori* in East Asia. In fact, recent discovery of J-Western *cagA* (Fig. 5) [38], [39], which is clearly distinct from authentic Western *cagA*, indicates that such missing *cagA* alleles could be found in isolated areas of East Asia in the future.

Now, the largest question that remains unsolved with CagA evolution is the origin of the right arm (D_R_) of the EPIYA-D segment, to which SHP2 binds with high-affinity. There could have existed yet another *H. pylori* lineage or another species in the stomach of ancient East Asian people that supplied a primordial *D_R_* sequence to *H. pylori* carrying Amerind *cagA* via intergenomic recombination. Alternatively, *D_R_* may have evolved from *C_R_* through accumulation of smaller mutations through mutagenesis and mutual homologous recombination. Lack of tandem amplification of a part of *cagA* through *CM*-mediated unequal recombination in Amerind and East Asian strains might have also accelerated diversification of the *D_R_* sequence from the *C_R_* sequence. Analysis of evolutionary pathways of C_R_ and D_R_ is difficult because of the short length of the segment, their low sequence identity, and the difference in their length (Fig. 1B). The short segment length makes it difficult to analyze them for recombination as carried out in the other parts. The low identity and the difference in their length make it difficult to relate them through simple accumulation of mutations.

Lack of the intact EPIYA-D segment in the New World indicates that *H. pylori* strains carrying Amerind CagA (or, possibly, J-Western CagA) but not East Asian CagA, did spread to the American continents through the Bering Strait together with ancient Asian people [37]. Although much less likely, an alternative idea will be that EPIYA-D was born after passing the Berling Strait, but most, if not all, of the ancient peoples who had carried *H. pylori* with EPIYA-D returned to repopulate East Asia for unknown reason. In any case, extensive sequence analysis of c*agA* genes isolated from indigenous peoples living in East Asia and American continents may shed light on the process of *cagA* gene diversification in the context of ancient human migrations.

### Adaptive evolution

The positively selected residues showed different patterns of diversification between the three phylogenetic groups of *cagA*. Association between the amino acid substitutions and phylogeny was found. This means that each amino acid was potentially selected at a different point in evolution, in a unique context, which may suggest that each is involved in a different function. Experimental analysis of these residues may provide a clue to the interactions between the CagA protein and humans.

*cagA* contains at least seven codons where an amino acid change was positively selected (Table 2) likely because of its effect on the function. The amino acid changes in residue 989 (in strain 26695) might reflect adaptive changes in the interaction of CagA with PAR1/MARK kinases in the East Asian groups different from the Western/J-Western group. We have not noticed any report on difference in these kinases between these two ethnic groups. If there be such a difference, it might indicate *Homo sapiens* – *H. pylori* co-evolution of some sort.

Also notably, residue 634 (in strain 26695) is located in the vicinity of the RxR motif (residues 619 and 621), which plays a critical role in the interaction of CagA with membrane phosphatidylserine (PS) [48]. Because the CagA-PS interaction is important for the CagA delivery into the host cells as well as membrane localization of the delivered CagA, the diversifying selection at residue 634 might be related to the interaction of CagA with this membrane phospholipid. PS is also known to be important in programmed cell death and immune response. Co-evolution between *H. pylori* lipopolysaccharide synthesis enzymes for Lewis antigen mimicry and human immune systems has been revealed (see, for example, a recent genome paper [43]). We do not know whether the diversity at residue 634 is related to the diversity in PS-related human genes, so we cannot discuss possible co-evolution between this bacterial oncogene and the genes related to this human phosholipid now. So far, no studies have reported the function of the other five residues, three of which are in the conserved regions.

Two recent studies reported sites for positively selected changes in *cagA* inferred by related statistical methods [49], [50] Compared with these studies, our methods are more selective. We cautiously explored the sites by codon-by-codon analysis and by filtering all the candidates with more than two gaps in the alignment (Materials and methods). More specifically, the “fixed-effects likelihood” model has three major advantages over PAML [51], [52]. First, it does not assume a distribution pattern of substitution rates in a gene, making the estimation of synonymous and non-synonymous substitution rates potentially more accurate. Particularly, it does not assume as PAML that synonymous substitution rates are constant for the entire length of a gene. This assumption would elevate rate of false positives when incorrect [53]. This is important in *cagA* gene that shows large difference in divergence along the gene (Fig. S5). Second, p-value is derived as a level of significance at every site. Although this likelihood method requires a larger sample size, it can thus control discovery rate at a desired level as we conducted in our study. Third, it is much more computationally efficient. It allowed us to conduct maximum-likelihood estimation and statistical tests for our very large data set (133 sequences in total).

## Materials and Methods

### *cagA* sequences

Amino acid sequences of CagA were retrieved from the NCBI protein database (http://www.ncbi.nlm.nih.gov/protein) and corresponding nucleotide sequences of *cagA* were retrieved from NCBI nucleotide database (http://www.ncbi.nlm.nih.gov/nuccore) as of August 17th, 2010. Entries without sequences of EPIYA segments were removed by searching for EPIYA segments using BLASTP [29]. Entries too short for assessing organization of EPIYA segments were omitted from analysis and are in Table S2.

### Sequence analysis

Sequence alignment was done by MUSCLE [54] and ClustalW [55]. Sequence logo was created by WebLogo version 2.8.2 [33]. Two CM sequences in the major Western type CagA and all the EPIYA motifs in non-chimeric segments were used for construction of the logo of CM sequences and EPIYA motifs, respectively.

### Detection of positively-selected amino acid changes

The coding sequences were translated to amino acid sequences and aligned using ClustalW. The aligned sequences were then replaced with the corresponding DNA sequences, preserving the gaps obtained during alignment of the amino acid sequences.

To detect potential positive selection we used the fixed-effects likelihood (FEL) model implemented in HyPhy [56]. According to the procedure, “two rate FEL”, the non-synonymous and synonymous substitutions rates (dN and dS) were directly estimated at each codon to accommodate site-by-site variation. Using a codon-based substitution model, based on the MG94 model [57] and augmented by the GTR model of nucleotide substitution, dN and dS were estimated by a maximum likelihood method, with and without the constraint that dN = dS. A likelihood ratio test was then conducted to assess whether dN was significantly different from dS.

Multiple alignment of *cagA* nucleotide sequences inevitably produces many gaps, particularly in its C-terminal sequences, which cause uncertainty in the inference of positive selection [58]. Therefore, we focused on stably aligned sequence regions in our large dataset. After filtering codons containing more than two gaps, we identified positively-selected sites with FDR (false discovery rate) adjusted to p-value<0.001. We confirmed that the seven identified sites were consistently found in nucleotide sequences aligned by another EINSI strategy in the MAFFT alignment package [59].

## Supporting Information

Figure S1 **All structural variants of CagA classified by recombination processes proposed for their formation.** (A) Major types. (B) Homologous recombination at CM sequence. (C) Recombination at *EPIYA* motif. (D) Illegitimate recombination by short sequence identity.
(PDF)

Figure S2 **Consensus sequences of CM/** ***CM*** **sequences and EPIYA/** ***EPIYA*** **motifs.** Upper: amino acid sequences. Lower: nucleotide sequences. (A) Left CM/*CM* sequence. (B) Right CM/*CM* sequence. (C) Western EPIYA-A/*EPIYA-A* motif. (D) Western EPIYA-B_C_/*EPIYA-B_C_* motif. (E) Western EPIYA-C/*EPIYA-C* motif. (F) East Asian EPIYA-A/*EPIYA-A* motif. (G) East Asian EPIYA-B_D_/*EPIYA-B_D_* motif. (H) East Asian EPIYA-D/*EPIYA-D* motif.
(PDF)

Figure S3 **Deletion involving the right CM sequence.** (A) Process of deletion and (B, C) sequence alignments. (D) Process of deletion at EPIYA-B_C_ and sequence alignment. Similar sequences presumed to be involved in recombination are boxed. Black: *EPIYA* motif. Gray: *CM* sequence.
(PDF)

Figure S4 **Additional cases of illegitimate recombination.** Inferred processes of illegitimate recombination and alignments in (A) MK M-03, MK M-05, (B) K192, (C) MK M-06, FGC146-1, F46, F18, (D) F92, (E) F56, (F) F26, K263, (G) F17, (H) MK F-02, (I) F75, (J) HPI-10, (K) G1050A, 1091, Hp51, (L) Shi470_1, (M) F65, (N) Alaska7, (O) K262, Hpcnic-27, (P) J-248, (Q) J-187, Z4, and (R) HN-91. Sequences presumed to be involved in the recombination are boxed. Black: *EPIYA* motif. Gray: *CM* sequence.
(PDF)

Figure S5 **Alignment of whole nucleotide sequence of cagA gene.**
(FASTA)

Table S1 **Sequence types of CagA with a recombination process proposed for their formation.**
(XLS)

Table S2 **Entries with sequences too short to classify all EPIYA segments.**
(XLS)

Table S3 **Sequences used for analysis of positive selection.**
(XLS)
