# Supplementary material for: Evolution of cagA Oncogene of Helicobacter pylori through Recombination
Source: PLoS One. 2011 Aug 11;6(8):e23499. doi: 10.1371/journal.pone.0023499 (PMC3154945; doi:10.1371/journal.pone.0023499)
Supplement: Figure S1 — All structural variants of CagA classified by recombination processes proposed for their formation. (A) Major types. (B) Homologous recombination at CM sequence. (C) Recombination at EPIYA motif. (D) Illegitimate recombination by short sequence identity. (PDF) [file pone.0023499.s001.pdf]

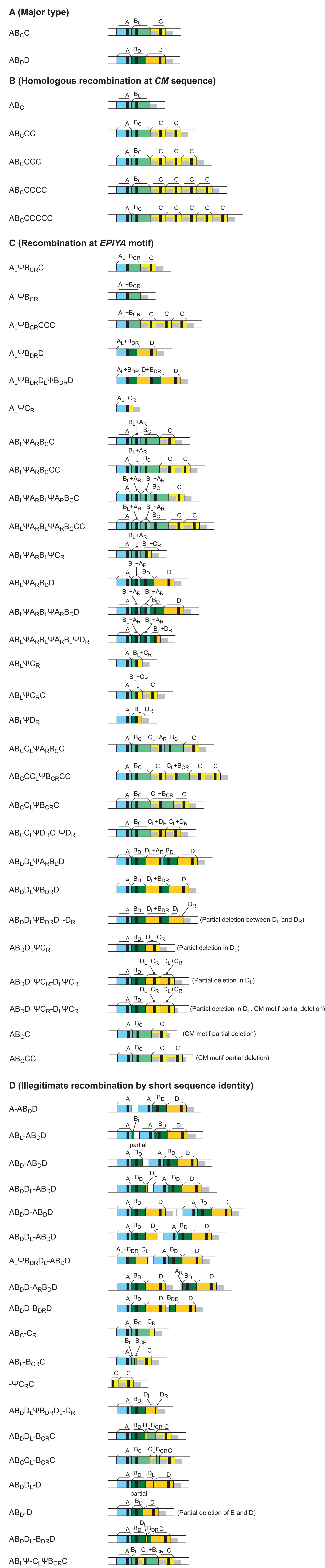

A (Major type)
AB_CC
AB_DD
B (Homologous recombination at CM sequence)
AB_C
AB_CCC
AB_CCCC
AB_CCCCC
AB_CCCCCC
C (Recombination at EPIYA motif)
$A_L\Psi B_{CR}C$
$A_L\Psi B_{CR}$
$A_L\Psi B_{CR}CCC$
$A_L\Psi B_{DR}D$
$A_L\Psi B_{DR}D_L\Psi B_{DR}D$
$A_L\Psi C_R$
$AB_L\Psi A_RB_CC$
$AB_L\Psi A_RB_CCC$
$AB_L\Psi A_RB_L\Psi A_RB_CC$
$AB_L\Psi A_RB_L\Psi A_RB_CCC$
$AB_L\Psi A_RB_L\Psi C_R$
$AB_L\Psi A_RB_DD$
$AB_L\Psi A_RB_L\Psi A_RB_DD$
$AB_L\Psi A_RB_L\Psi A_RB_L\Psi D_R$
$AB_L\Psi C_R$
$AB_L\Psi C_RC$
$AB_L\Psi D_R$
$AB_CC_L\Psi A_RB_CC$
$AB_CCC_L\Psi B_{CR}CC$
$AB_CC_L\Psi B_{CR}C$
$AB_CC_L\Psi D_RC_L\Psi D_R$
$AB_DD_L\Psi A_RB_DD$
$AB_DD_L\Psi B_{DR}D$
$AB_DD_L\Psi B_{DR}D_L-D_R$
(Partial deletion between D_L and D_R)
$AB_DD_L\Psi C_R$
(Partial deletion in D_L)
$AB_DD_L\Psi C_R-D_L\Psi C_R$
(Partial deletion in D_L)
$AB_DD_L\Psi C_R-D_L\Psi C_R$
(Partial deletion in D_L, CM motif partial deletion)
AB_CC
(CM motif partial deletion)
AB_CCC
(CM motif partial deletion)
D (Illegitimate recombination by short sequence identity)
$A-AB_DD$
AB_L-AB_DD
AB_D-AB_DD
$AB_DD_L-AB_DD$
AB_DD-AB_DD
$AB_DD_L-AB_DD$
$A_L\Psi B_{DR}D_L-AB_DD$
$AB_DD-A_RB_DD$
$AB_DD-B_{DR}D$
AB_C-C_R
$AB_L-B_{CR}C$
$-\Psi C_RC$
$AB_DD_L\Psi B_{DR}D_L-D_R$
$AB_DD_L-B_{CR}C$
$AB_CC_L-B_{CR}C$
AB_DD_L-D
AB_D-D
(Partial deletion of B and D)
$AB_DD_L-B_{DR}D$
$AB_L\Psi-C_L\Psi B_{CR}C$
