# Supplementary material for: Evolution of cagA Oncogene of Helicobacter pylori through Recombination
Source: PLoS One. 2011 Aug 11;6(8):e23499. doi: 10.1371/journal.pone.0023499 (PMC3154945; doi:10.1371/journal.pone.0023499)
Supplement: Figure S2 — Consensus sequences of CM/ CM sequences and EPIYA/ EPIYA motifs. Upper: amino acid sequences. Lower: nucleotide sequences. (A) Left CM/CM sequence. (B) Right CM/CM sequence. (C) Western EPIYA-A/EPIYA-A motif. (D) Western EPIYA-BC/EPIYA-BC motif. (E) Western EPIYA-C/EPIYA-C motif. (F) East Asian EPIYA-A/EPIYA-A motif. (G) East Asian EPIYA-BD/EPIYA-BD motif. (H) East Asian EPIYA-D/EPIYA-D motif. (PDF) [file pone.0023499.s002.pdf]

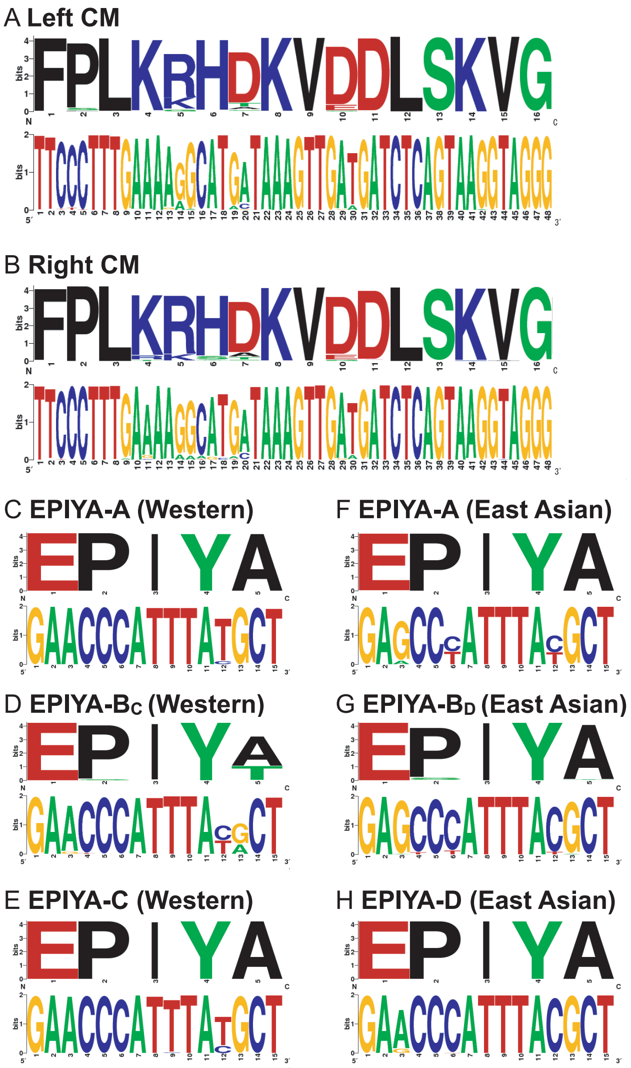
A Left CM
B Right CM
C EPIYA-A (Western)
D EPIYA-BC (Western)
E EPIYA-C (Western)
F EPIYA-A (East Asian)
G EPIYA-BD (East Asian)
H EPIYA-D (East Asian)
