# Supplementary figures and images for: Evolution of cagA Oncogene of Helicobacter pylori through Recombination
Source: PLoS One. 2011 Aug 11;6(8):e23499. doi: 10.1371/journal.pone.0023499 (PMC3154945; doi:10.1371/journal.pone.0023499)

A

26695

Hpcnic47, Hp93, Hp72,  
259c4, Hp152944,  
Hp140676, PZ5081, v225d

B (deletion pattern except for v225d)

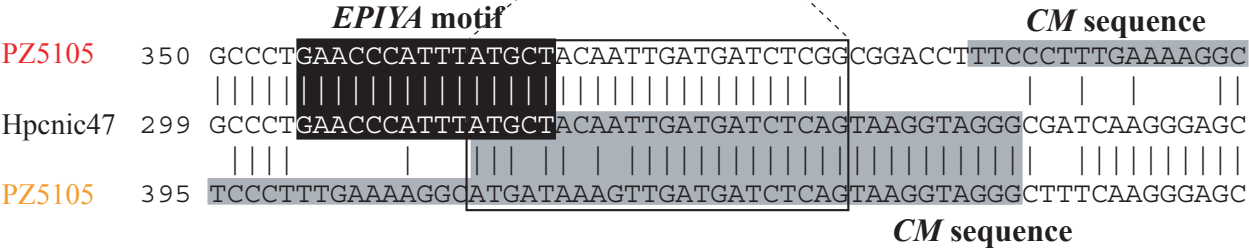

C (deletion pattern of v225d)

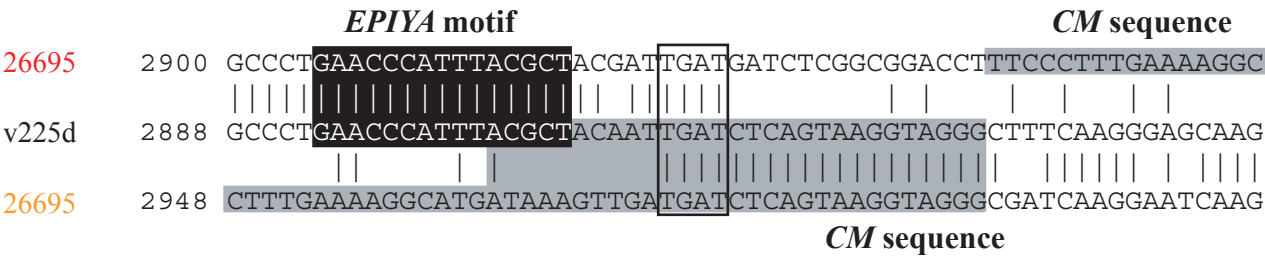

D

Colombia  
NA1994

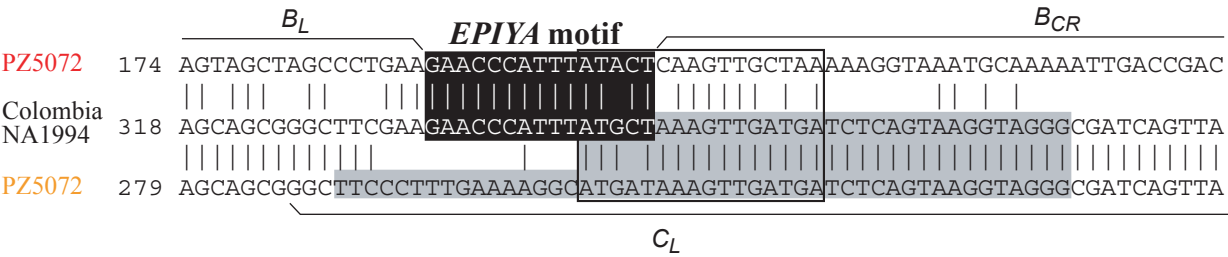

Supplement: Figure S3 — Deletion involving the right CM sequence. (A) Process of deletion and (B, C) sequence alignments. (D) Process of deletion at EPIYA-BC and sequence alignment. Similar sequences presumed to be involved in recombination are boxed. Black: EPIYA motif. Gray: CM sequence. (PDF) [file pone.0023499.s003.pdf]

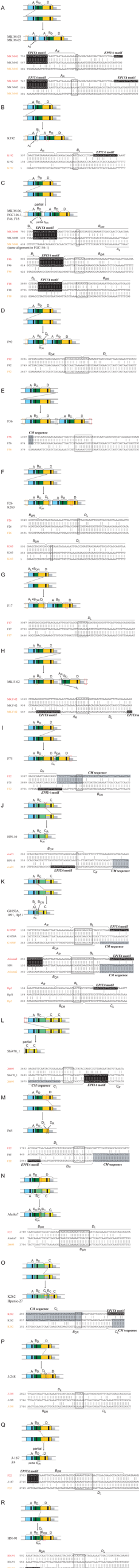

Supplement: Figure S4 — Additional cases of illegitimate recombination. Inferred processes of illegitimate recombination and alignments in (A) MK M-03, MK M-05, (B) K192, (C) MK M-06, FGC146-1, F46, F18, (D) F92, (E) F56, (F) F26, K263, (G) F17, (H) MK F-02, (I) F75, (J) HPI-10, (K) G1050A, 1091, Hp51, (L) Shi470_1, (M) F65, (N) Alaska7, (O) K262, Hpcnic-27, (P) J-248, (Q) J-187, Z4, and (R) HN-91. Sequences presumed to be involved in the recombination are boxed. Black: EPIYA motif. Gray: CM sequence. (PDF) [file pone.0023499.s004.pdf]
